# Anal Cancer Incidence Rates Among Men and Women With and Without HIV in South Africa

**DOI:** 10.1093/ofid/ofaf537

**Published:** 2025-09-01

**Authors:** Nathalie Verónica Fernández Villalobos, Yann Ruffieux, Chido Chinogurei, Andreas D Haas, Nicola Low, Matthias Egger, Jenni Noble, Gary Maartens, Naomi Folb, Eliane Rohner

**Affiliations:** Institute of Social and Preventive Medicine, University of Bern, Bern, Switzerland; Institute of Social and Preventive Medicine, University of Bern, Bern, Switzerland; Centre for Integrated Data and Epidemiological Research, School of Public Health, University of Cape Town, Cape Town, South Africa; Institute of Social and Preventive Medicine, University of Bern, Bern, Switzerland; Centre for Integrated Data and Epidemiological Research, School of Public Health, University of Cape Town, Cape Town, South Africa; Institute of Social and Preventive Medicine, University of Bern, Bern, Switzerland; Centre for Integrated Data and Epidemiological Research, School of Public Health, University of Cape Town, Cape Town, South Africa; Department of Infectious Diseases and Hospital Epidemiology, University Hospital Zurich, University of Zurich, Zurich, Switzerland; Population Health Sciences, Bristol Medical School, University of Bristol, Bristol, UK; Medscheme, Cape Town, South Africa; Division of Clinical Pharmacology, Department of Medicine, University of Cape Town, Cape Town, South Africa; Medscheme, Cape Town, South Africa; Division of Clinical Pharmacology, Department of Medicine, University of Cape Town, Cape Town, South Africa; Institute of Social and Preventive Medicine, University of Bern, Bern, Switzerland

**Keywords:** anus neoplasms, HIV, risk factors, South Africa

## Abstract

**Background:**

More than 7.5 million people in South Africa have HIV, but little is known about the association of HIV and anal cancer incidence. We examined anal cancer incidence in a large South African cohort of insured men and women.

**Methods:**

We conducted a cohort study using reimbursement claims data from a South African medical insurance scheme (01/2011-07/2020) to assess anal cancer rates among people with and without HIV aged ≥18 years. We estimated adjusted hazard ratios (aHRs) for the association of HIV and incident anal cancer using flexible parametric survival models. Covariates included sex, age, calendar year, a history of genital warts and other sexually transmitted infections, and in women, cervical precancer.

**Results:**

We included 1 068 915 people of whom 69 985 (7%) were living with HIV. Over 3 933 145 person-years, 122 anal cancers were diagnosed (crude rate: 3.1/100 000 person-years; 95% confidence intervals [CI] 2.6–3.7). People with HIV had a 4-fold higher anal cancer risk than people without HIV (aHR 4.43; 95% CI 2.44–8.04). While anal cancer rates were similar among men and women, older age (≥65 vs 45–54 years; aHR 5.01; 95% CI: 2.94–8.53), a history of genital warts (aHR 7.56; 95% CI: 2.28–25.07), and among women, a prior cervical precancer diagnosis (aHR 5.70; 95% CI 1.75–18.58) were associated with a higher anal cancer risk.

**Conclusions:**

In South Africa, men and women with HIV, older individuals, people with a history of genital warts, and women with a prior cervical precancer diagnosis might benefit from prioritized access to anal cancer screening.

## Teaser

We assessed anal cancer rates among people with and without HIV in South Africa. Risk was higher among people with HIV, older people, those with genital warts, and women with prior cervical precancer, supporting targeted screening in these priority groups.

Anal carcinoma, while rare [1], has shown a concerning rise in incidence rates in high income countries over time [2]. The reasons for this increase are not fully understood. Anal squamous cell carcinomas (SCCs) are the most common histological subtype, followed by adenocarcinomas [3]. Anal SCCs have been causally linked to high-risk human papillomavirus (HPV) infection, with a meta-analysis reporting an HPV prevalence of 84% in anal carcinomas [4]. In South Africa, anal cancer rates rose among women and men between 1994 and 2012, likely driven by the country's high HPV and HIV prevalence [5]. From 2011 to 2021, the incidence of anal SCC continued to increase, making it one of the fastest rising HPV-related cancers in South Africa, with annual increases of more than 8% among both men and women [6].

People with HIV (PWH), especially those with advanced immunodeficiency [7], have higher anal cancer rates than the general population [8]. Globally, around 21% of men and 3% of women with anal SCC have HIV, with notable regional variation [9]. Most men with anal cancer and HIV live in high-income regions such as Europe and North America, where anal intercourse among men who have sex with men (MSM) contributes substantially to both HIV and HPV transmission [9]. In sub-Saharan Africa, the epicenter of the HIV epidemic, an estimated 1 in 3 men and 1 in 4 women with anal SCC live with HIV [9, 10]. Specifically, the Johannesburg Cancer Study, which enrolled Black African people diagnosed with cancer at 3 different tertiary hospitals between 1995 and 2016, found that among 115 individuals with an anal cancer diagnosis 31% lived with HIV [11].

HPV-induced anal carcinogenesis appears to follow a stepwise progression from high-risk HPV infection to anal intraepithelial neoplasia and cancer [12, 13]. The Anal Cancer–HSIL Outcomes Research (ANCHOR) trial in the United States (US) showed that screening for and treatment of anal high-grade lesions can reduce anal cancer risk among PWH [14]. While routine screening is not recommended for the general population due to the rarity of the disease, the International Anal Neoplasia Society's consensus guidelines recommend targeted screening for selected priority groups, including PWH, MSM, or women with HPV-related precancers [15–18]. To date, no dedicated anal cancer screening guidelines are available for South Africa and data on anal cancer incidence and associated risk factors in sub-Saharan Africa are scarce. We examined the association of HIV status and other potential risk factors with incident anal cancer diagnosis among insured individuals in South Africa.

## METHODS

### Study Design and Data Source

We analyzed reimbursement claims data from a South African medical insurance scheme from 2011 to 2020. The insurance scheme manages healthcare through services such as disease and case management, while also handling key administrative operations such as processing claims, enrolling members, managing billing, and facilitating communication. It partners with a wide network of healthcare providers including hospitals, doctors, and other facilities. The claims database included codes based on the International Classification of Diseases (ICD)-10 and the Anatomical Therapeutic Chemical (ATC) Classification System. The Human Research Ethics Committee of the University of Cape Town and the Cantonal Ethics Committee of the Canton of Bern approved the analysis of these data.

### Inclusion Criteria and Definitions

We included people aged ≥18 years who were covered by the medical insurance scheme at any time between January 1, 2011 and July 1, 2020. We identified PWH based on the following HIV indicators: ATC codes indicating antiretroviral therapy (ART), HIV-related ICD-10 diagnoses (B20–24, F02.4, O98.7, R75, Z21), HIV-related laboratory tests (positive HIV test, HIV RNA viral load, CD4 cell count), and enrolment in the Aid for AIDS disease management program. We assigned a positive HIV status to people with ≥2 HIV indicators, and a negative HIV status to people with no HIV indicator. People with a single HIV indicator were excluded from the main analysis to increase the specificity of our definition.

Our outcome of interest was incident anal cancer, which we defined as ≥2 claims with ICD-10 codes for anal cancer (C21) recorded in a person's inpatient or outpatient reimbursement claims. We included all anal cancer diagnoses, irrespective of histology, as information on histological subtype was not consistently available. We excluded people with a single C21 code. For PWH, time-at-risk started at the date of their first HIV indicator, their 18th birthday, or January 1, 2011, whichever came last. For people without HIV, time-at-risk started at the enrolment date into the medical insurance scheme, their 18th birthday, or January 1, 2011, whichever came last. For all included individuals, time-at-risk ended at the first diagnosis of anal cancer, transfer from the medical insurance scheme, death, or database closure (July 1, 2020), whichever came first. We excluded people with an anal cancer diagnosis at the start of or before their time-at-risk.

We used ICD-10 codes to define the following exposures of interest: genital warts (A63.0), other sexually transmitted infections (STIs) including syphilis (A51-A53), gonorrhoea (A54), chlamydia (A55, A56), chancroid (A57), granuloma inguinale (A58), trichomoniasis (A59), anogenital herpes simplex infection (A60), other specified predominantly sexually transmitted diseases (A63.8), and unspecified sexually transmitted diseases (A64), and among women, cervical precancer (N87.1, N87.2, and D06).

### Statistical Methods

We used descriptive statistics to assess sociodemographic characteristics by HIV status and by sex. We calculated crude anal cancer incidence rates per 100 000 person-years by dividing the number of individuals with an incident anal cancer diagnosis by the total number of person-years at risk.

We fit Royston–Parmar flexible parametric survival models to estimate anal cancer incidence rates as a continuous function of age (4 degrees of freedom) and calendar year (2 degrees of freedom) [19]. We included either HIV status or sex as a predictor in these models and included an interaction between these factors and the time scale (age or calendar year). We used Royston–Parmar survival models on the follow-up scale to estimate unadjusted and adjusted hazard ratios (aHRs) for the association between anal cancer incidence and different risk factors. Risk factors of interest included HIV status (negative/positive), sex (female/male), age (18–34, 35–44, 45–54, 55–64, ≥ 65 years, time-updated), history of genital warts (no/yes, time-updated), history of other STIs (no/yes, time-updated), and calendar year (2011–2013, 2014–2016, 2017–2020, time-updated). Additionally, we adjusted the multivariable models for self-identified population group (Black African, other, unknown). We tested for an interaction between sex and HIV status. Among women, we estimated unadjusted and adjusted HRs for the association of a history of cervical precancer (no/yes, time-updated) with anal cancer. We report summary HRs based on models that assume proportional hazards for all risk factors. We also modeled interactions between follow-up time and each risk factor and graphically display the HRs over time. In all models, we chose the number of degrees of freedom for baseline hazards using the Akaike Information Criteria. All interactions with the time scale were modeled with 1 degree of freedom. Analyses were performed using R 4.2.3 (R Foundation for Statistical Computing, Vienna, Austria).

### Sensitivity Analyses

We performed sensitivity analyses to assess the impact of our definition of PWH: (1) we extended the time-at-risk among PWH to start 1 or 2 years before their first HIV indicator, and (2) we included people with a single HIV indicator as PWH. Additionally, we changed our definition of anal cancer to include people with a single C21 code.

## RESULTS

### Study Population

Of 1 549 123 people covered by the medical insurance scheme at any time between January 1, 2011 and July 1, 2020, we included 1 068 915 (69%). We excluded 19 152 people with missing information on sex or date of birth. Additionally, we excluded 461 056 people for the reasons detailed in Supplementary Figure 1. Of the included 1 068 915 individuals, 7% were PWH (*n* = 69 985; Table 1). The percentage of women was slightly higher among PWH (58%, *n* = 40 522) than people without HIV (52%, *n* = 517 195). The median age at the start of time-at-risk was 39.7 years (interquartile range [IQR] 33.4–47.3) in PWH and 36.3 years (IQR 26.1–49.9) in people without HIV. Genital warts and other STIs were more frequently diagnosed in PWH than in people without HIV. Participant characteristics by sex are shown in Supplementary Table 1.

**Table 1.** Participant Characteristics by HIV Status

	People Without HIV	People With HIV	Overall
	*N* = 998 930	*N* = 69 985	*N* = 1 068 915
Female	517 195 (52%)	40 522 (58%)	557 717 (52%)
Median age at baseline^a^ [IQR] (y)	36.3 [26.1, 49.9]	39.7 [33.4, 47.3]	36.7 [26.7, 49.6]
Age group at baseline^a^ (y)			
18–34	470 264 (47%)	21 610 (31%)	491 874 (46%)
35–44	194 324 (20%)	26 181 (37%)	220 505 (21%)
45–54	163 785 (16%)	16 267 (23%)	180 052 (17%)
55–64	101 699 (10%)	5306 (8%)	107 005 (10%)
≥ 65	68 858 (7%)	621 (1%)	69 479 (7%)
Population group			
Black African	495 005 (50%)	61 157 (88%)	556 162 (52%)
Other	313 595 (31%)	2232 (3%)	315 827 (30%)
Unknown	190 330 (19%)	6596 (9%)	196 926 (18%)
Baseline year^a^			
2011–2013	575 041 (58%)	33 133 (47%)	608 174 (57%)
2014–2016	157 160 (16%)	16 825 (24%)	173 985 (16%)
2017–2020	266 729 (27%)	20 027 (29%)	286 756 (27%)
Genital warts^b^	3007 (<1%)	1892 (3%)	4899 (<1%)
Other STIs^b^	22 111 (2%)	6163 (9%)	28 274 (3%)

The results are reported as numbers and percentages if not otherwise stated.

^a^Baseline refers to the start of time-at-risk.

^b^Diagnosis during or before time-at-risk.

IQR, interquartile range; STI, sexually transmitted infection.

### Incident Anal Cancer Diagnosis

During 3 933 145 person-years, 122 incident anal cancer diagnoses were recorded (crude incidence: 3.1/100 000 person-years; 95% confidence intervals [CI] 2.6–3.7). Of 122 people with anal cancer, 67 were women (55%) and 18 had HIV (15%; Table 2). The anal cancer incidence rate in PWH was 6.6 per 100 000 person-years (95% CI 3.9–10.4). The median age at anal cancer diagnosis was 44.0 years in PWH (IQR 42.9–57.4) and 66.3 years in people without HIV (IQR 54.4–72.8; Table 3).

**Table 2.** Characteristics of Individuals With or Without Incident Anal Cancer

	No Anal Cancer	With Anal Cancer	Overall
	*N* = 1 068 793	*N* = 122	*N* = 1 068 915
Female	557 650 (52%)	67 (55%)	557 717 (52%)
People with HIV	69 967 (7%)	18 (15%)	69 985 (7%)
Median age at baseline^a^ [IQR] (y)	36.7 [26.7, 49.6]	59.2 [47.3, 68.4]	36.7 [26.7, 49.6]
Age group at baseline^a^ (y)			
18–34	491 866 (46%)	8 (7%)	491 874 (46%)
35–44	220 486 (21%)	19 (16%)	220 505 (21%)
45–54	180 025 (17%)	27 (22%)	180 052 (17%)
55–64	106 985 (10%)	20 (16%)	107 005 (10%)
≥ 65	69 431 (7%)	48 (39%)	69 479 (7%)
Population group			
Black African	556 130 (52%)	32 (26%)	556 162 (52%)
Other	315 773 (30%)	54 (44%)	315 827 (30%)
Unknown	196 890 (18%)	36 (30%)	196 926 (18%)
Baseline year^a^			
2011–2013	608 087 (57%)	87 (71%)	608 174 (57%)
2014–2016	173 970 (16%)	15 (12%)	173 985 (16%)
2017–2020	286 736 (27%)	20 (16%)	286 756 (27%)
Genital warts^b^	4896 (<1%)	3 (3%)	4899 (<1%)
Other STIs^b^	28 271 (3%)	3 (3%)	28 274 (3%)

The results are reported as numbers and percentages if not otherwise stated.

^a^Baseline refers to the start of time-at-risk.

^b^Diagnosis during or before time-at-risk.

IQR, interquartile range; STI, sexually transmitted infection.

**Table 3.** Characteristics of Participants Diagnosed With Anal Cancer, at the Time of Their Diagnosis, by HIV Status

	People Without HIV	People With HIV	Overall
	*N* = 104	*N* = 18	*N* = 122
Female	56 (54%)	11 (61%)	67 (55%)
Median age [IQR] (y)	66.3 [54.4, 72.8]	44.0 [42.9, 57.4]	61.9 [50.7, 71.9]
Age group (y)			
18–34	4 (4%)	1 (6%)	5 (4%)
35–44	5 (5%)	9 (50%)	14 (12%)
45–54	18 (17%)	3 (17%)	21 (17%)
55–64	22 (21%)	4 (22%)	26 (21%)
≥65	55 (53%)	1 (6%)	56 (46%)
Calendar year			
2011–2013	22 (21%)	1 (6%)	23 (19%)
2014–2016	30 (29%)	4 (22%)	34 (28%)
2017–2020	52 (50%)	13 (72%)	65 (53%)
History of genital warts	0	3 (17%)	3 (3%)
History of other STIs	0	3 (17%)	3 (3%)

The results are reported as numbers and percentages if not otherwise stated.

IQR, interquartile range; STI, sexually transmitted infection.

**Figure 1.**
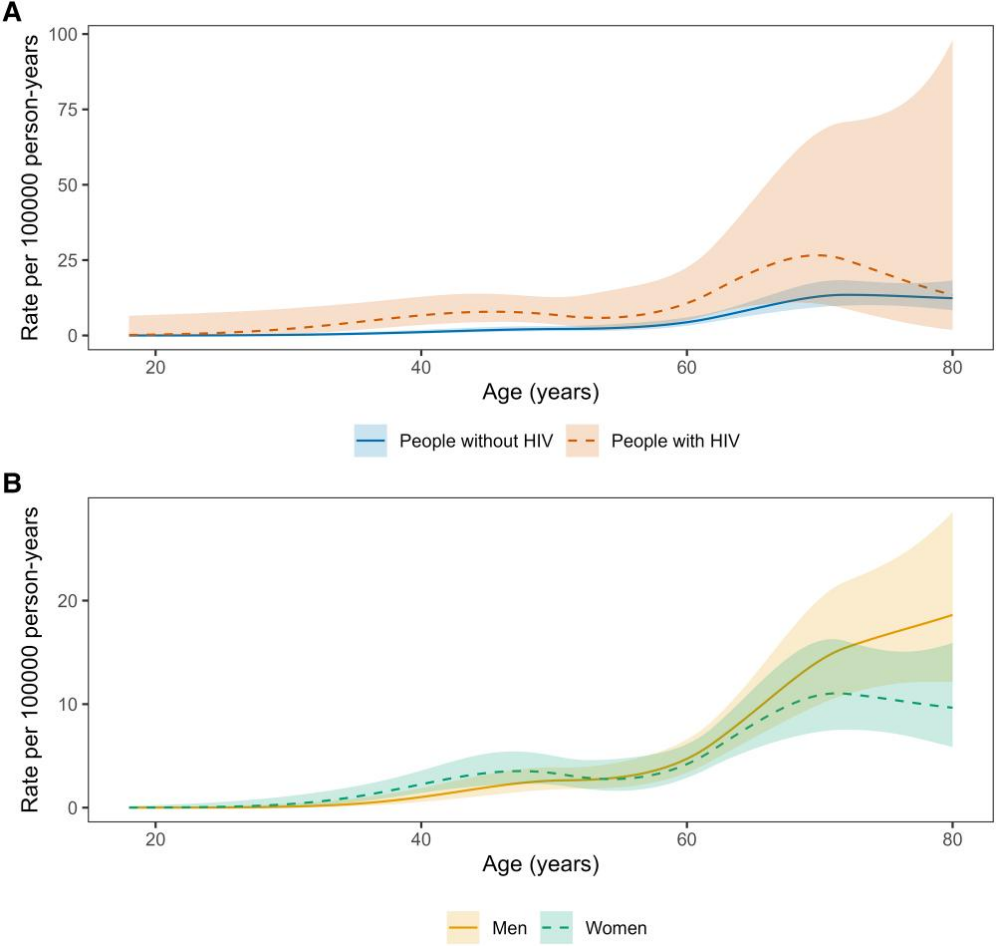
Incidence rates of anal cancer by (*A*) age and HIV status, and (*B*) age and sex.

**Figure 2.**
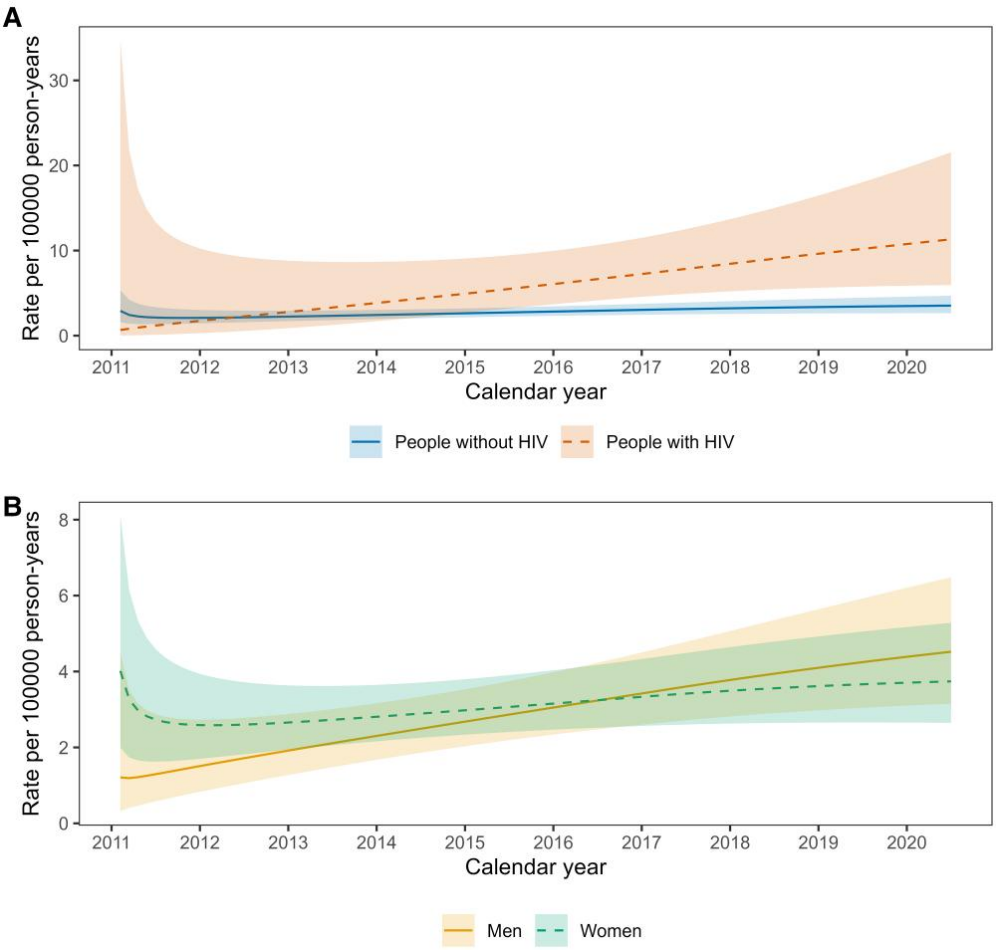
Incidence rates of anal cancer by (*A*) calendar year and HIV status and (*B*) calendar year and sex.

Anal cancer incidence rates increased with older age and were higher among PWH than people without HIV across the age range (Figure 1*A*). Age-specific anal cancer rates were similar in men and women (Figure 1*B*). Over calendar time, anal cancer incidence rates increased among PWH, but were stable among people without HIV (Figure 2*A*). Women tended to have higher anal cancer rates than men in the earlier calendar years (Figure 2*B*).

### Risk Factors for Developing Anal Cancer

PWH had a 4-fold higher risk of anal cancer than people without HIV (aHR 4.43; 95% CI 2.44–8.04; Table 4). Anal cancer rates were similar in women compared with men (aHR 0.97; 95% CI 0.68–1.38) with no evidence for an interaction between HIV status and sex (*P*-value: 0.51). The risk of anal cancer increased with age (Table 4). People with a previous diagnosis of genital warts had a substantially higher anal cancer risk (aHR 7.56; 95% CI: 2.28–25.07) than those without genital warts. We did not find a clear association between a history of other STIs and the risk of anal cancer (aHR 1.46; 95% 0.44–4.80). Supplementary Figure 2 shows the HRs from the adjusted model as a function of follow-up time when relaxing the proportional hazards assumption. Four out of 67 women with anal cancer had a prior cervical precancer diagnosis recorded (Supplementary Table 2). Women with a prior diagnosis of cervical precancer had an almost 6-fold higher risk of an incident anal cancer diagnosis than those without cervical precancer (aHR 5.70; 95% CI 1.75–18.58; Table 4).

**Table 4.** Hazard Ratios for Factors Potentially Associated With Incident Anal Cancer. The Adjusted Models Control for the Risk Factors Listed in the Table and for Population Group

	Both Sexes Included		Only Women Included	
	Unadjusted	Adjusted	Unadjusted	Adjusted
HIV status				
Negative	1	1	1	1
Positive	2.28 (1.38–3.77)	4.43 (2.44–8.04)	2.32 (1.21–4.42)	4.03 (1.81–9.00)
Sex				
Male	1	1	…	…
Female	1.09 (0.76–1.55)	0.97 (0.68–1.38)	…	…
Age^a^ (y)				
18–34	0.15 (0.06–0.40)	0.17 (0.06–0.46)	0.27 (0.09–0.77)	0.30 (0.10–0.88)
35–44	0.62 (0.31–1.21)	0.60 (0.31–1.19)	0.85 (0.36–1.99)	0.76 (0.32–1.80)
45–54	1	1	1	1
55–64	1.72 (0.97–3.06)	1.82 (1.02–3.25)	1.69 (0.76–3.78)	1.87 (0.83–4.22)
≥65	5.04 (3.05–8.33)	5.01 (2.94–8.53)	4.36 (2.17–8.77)	4.53 (2.17–9.46)
Calendar year^a^				
2011–2013	1	1	1	1
2014–2016	1.40 (0.80–2.45)	1.47 (0.82–2.62)	1.63 (0.82–3.25)	1.59 (0.78–3.21)
2017–2020	2.01 (1.18–3.42)	1.75 (1.00–3.06)	1.73 (0.90–3.35)	1.29 (0.65–2.56)
Genital warts^a^				
No	1	1	1	1
Yes	6.03 (1.90–19.17)	7.56 (2.28–25.07)	6.59 (1.61–27.03)	4.01 (0.81–19.82)
Other STIs^a^				
No	1	1	1	1
Yes	0.98 (0.30–3.16)	1.46 (0.44–4.80)	0.89 (0.12–6.44)	0.93 (0.12–7.04)
Cervical precancer^a^				
No	…	…	1	1
Yes	…	…	6.02 (2.17–16.70)	5.70 (1.75–18.58)

^a^Time-updated variables.

STI: sexually transmitted infection.

### Sensitivity Analyses

When varying our definition of PWH and their time-at-risk, results remained broadly similar (Supplementary Tables 3 and 4). When including individuals with a single HIV indicator (*n* = 12 701) in our definition of PWH, the positive association between HIV and incident anal cancer diagnosis became slightly weaker (aHR 3.88; 95% CI 2.15–7.01; Supplementary Table 5). Similarly, when relaxing our anal cancer definition to only require 1 C21 ICD-10 code (105 additional diagnoses), we also found a weaker positive association between HIV and anal cancer risk (aHR 2.91; 95% CI 1.82–4.66; Supplementary Table 6) compared with the main analysis.

## DISCUSSION

We found that anal cancer diagnosis rates in this South African medical insurance cohort were 4 times higher in PWH than people without HIV, with similar rates in men and women. Anal cancer rates increased between 2011 and 2020 among PWH but not among people without HIV. While older age and a prior diagnosis of genital warts were associated with an increased anal cancer risk, a history of other STIs showed no clear association with incident anal cancer. Among women, anal cancer diagnosis rates were almost 6 times higher among those with a prior diagnosis of cervical precancer.

Our study is one of few examining anal cancer rates and associated risk factors in sub-Saharan Africa, using a large cohort of over 1 million men and women. However, there are some limitations to consider. Our study population consisted of people covered by a private-sector insurance scheme, who may differ substantially from the general South African population in terms of health care access, socioeconomic status, age, and population group structure, and also had a substantially lower HIV prevalence. Therefore, our findings, particularly the absolute risk estimates, might not be generalizable to the public sector or the general South African population. However, the relative associations between risk factors and incident anal cancer are likely to be more generalizable, as they reflect internal comparisons. Histological information was inconsistently available and, therefore, we could not perform stratified analyses for anal SCC and adenocarcinoma separately. Moreover, distinguishing distal rectal adenocarcinomas with local spread from primary anal adenocarcinomas can be difficult, and some misclassification cannot be ruled out. We were unable to account for lifestyle and behavioral factors such as smoking and sexual practices that could influence the relationship between HIV and incident anal cancer. In particular, we were unable to perform a subgroup analysis for MSM. In general, using claims data to define risk factors and anal cancer diagnoses may have led to some misclassification. To reduce the likelihood of false-positive anal cancer diagnoses, we required at least 2 C21 ICD-10 codes for an anal cancer diagnosis. However, this may have resulted in an underestimation of the anal cancer rate in our study. We categorized individuals as HIV-negative if there were no indicators of HIV, but some individuals may have had undiagnosed HIV. Furthermore, we did not have information on the timing of HIV infection and some individuals could have been living with HIV for a significant period before their first HIV indicator. However, sensitivity analyses that assumed an earlier HIV infection date yielded broadly similar results.

We and others [8, 11, 20] found that HIV infection was associated with an elevated anal cancer risk. This might partly be explained by higher anal HPV prevalence among PWH than those without HIV [21]. For example, the Johannesburg Cancer Study reported 2-fold increased odds of HIV among those diagnosed with anal cancer compared with infection-unrelated cancers [11]. Interestingly, the HIV prevalence among people with anal cancer in our study was substantially lower than that reported by the Johannesburg Cancer Study (31%) and other studies from South Africa [9, 11, 22]. While some misclassification of PWH as HIV-negative may have occurred in our study, the discrepancy is more likely mainly attributable to differences in the underlying study populations and anal cancer case definitions. The Johannesburg Cancer Study included only Black African individuals with cancer—a group with high HIV prevalence—whereas only half of our study population self-identified as Black Africans. Furthermore, the overall HIV prevalence in our privately insured cohort was substantially lower than that of the general South African population. A study from a tertiary hospital, which found an HIV prevalence of 80% among individuals with anal cancer, was conducted in KwaZulu Natal—the province with the highest HIV prevalence in South Africa—and was restricted to anal SCCs only. The differences in study populations and anal cancer case definitions may also partly explain why both the estimated anal cancer rates among PWH and the association with HIV in our study were lower than what others have reported [8, 17, 20]. To date, most studies on anal cancer incidence have been conducted in high-income countries outside of sub-Saharan Africa [17], where the HIV epidemics are largely concentrated among key populations, such as MSM. In contrast, South Africa faces a generalized HIV epidemic, predominantly driven by heterosexual transmission. Moreover, differences in health system infrastructure and access to diagnostic services may also contribute to the observed differences across studies. Interestingly, a recently published study from the South African National Cancer Registry found a low age-standardized anal cancer incidence rate of <1 per 100 000 person-years [6]. Of note, the National Cancer Registry is pathology-based and clinically diagnosed cases are not captured.

We found that anal cancer rates among PWH tended to increase over time, while they remained relatively stable among people without HIV. Interestingly, anal cancer rates have been rising in most countries over time [2, 23], including South Africa [5, 6]. For South Africa, it has been suggested that this increase in the general population could be due to high prevalence of cervical HPV infection in women, high HIV prevalence, and improved survival of PWH on ART, which allows precancerous lesions to progress to anal cancer [5, 6]. Among PWH, mixed anal cancer trends have been observed. An Australian study reported a continuous rise in anal cancer incidence rates among PWH between 1982 and 2012 [24]. In contrast, a US study analyzing data from 1996 to 2012 found that anal cancer incidence rates among PWH plateaued between 2000 and 2008 and declined thereafter [20]. Another US study found an annual decrease in anal cancer incidence of 2% among PWH between 2000 and 2016, which might be explained by the wide use of ART and early anal cancer screening guidelines [25].

In line with other studies [17, 26, 27], we found that the risk of developing anal cancer increased with older age. The International Anal Neoplasia Society's consensus guidelines suggest starting anal cancer screening at the age of 35 years for MSM and transgender women with HIV, while for other PWH screening is recommended starting at the age of 45 years [16]. Of note, in our study, 19 out of 122 individuals (10 with HIV and 9 without HIV) were diagnosed with anal cancer before the age of 45 years. A history of genital warts was also associated with a substantially higher anal cancer risk in our analysis. This result is in line with a Danish study among almost 50 000 individuals with genital warts, which found an increased risk of developing HPV-related anogenital and head and neck cancers in this population [28]. The reasons why individuals with anogenital warts have increased anal cancer rates are not fully understood, as anogenital warts are typically caused by nononcogenic low-risk HPV genotypes, such as HPV 6 and 11. Confounding by sexual behavioral may play a role as higher sexual activity and anal sex increase the likelihood of contracting both low-risk and high-risk HPV genotypes [29]. Interestingly, we did not find a clear association between a prior diagnosis of other STIs and anal cancer. Other behavioral factors such as smoking, for example, may also confound this association. Additionally, immunological factors may predispose individuals to persistent HPV infection and subsequent development of HPV-related diseases [28].

Consistent with our findings, other studies have shown that women with a history of cervical precancer face a higher risk of developing anal cancer than women in the general population [30–32]. High genotype-specific concordance between anal and cervical high-risk HPV infections indicates a common source of infection, such as vaginal and anal intercourse with the same infected partner, or transmission between the 2 sites through nonpenetrative sex or self-inoculation via fingers or other means [33]. Women with persistent anogenital high-risk HPV infections may experience immune dysregulation and an insufficient immune response to the virus resulting in a higher risk of HPV persistence and spread to other anogenital regions [31]. A collaborative pooled analysis of 36 studies including 13 427 women with paired cervical and anal samples suggested that HPV-based cervical cancer screening programs could help identify women at high risk for anal cancer, as especially older HIV-negative women with cervical HPV 16 had a similarly high anal cancer risk profiles as women with HIV [10].

While anal cancer screening is not recommended for the general population, the International Anal Neoplasia Society's consensus guidelines propose targeted screening for certain individuals including PWH, MSM, solid organ transplant recipients, and women with a history of HPV-related precancers [16]. Recent findings from the ANCHOR trial have shown that treating anal high-grade squamous intraepithelial lesions can substantially reduce the risk of developing anal cancer among PWH [14]. A survey examining anal screening recommendations among healthcare providers across 21 countries found high consensus on using anal cytology and digital anorectal examination as preferred screening methods but low consensus on other screening aspects such as the screening initiation age [34]. Notably, only Kenya was represented from the African region in this survey. No dedicated anal cancer screening guidelines for South Africa are currently available. Given the limited coverage of other cancer screening programs in South Africa, the feasibility of implementing a national anal cancer screening program warrants careful consideration. However, targeted screening approaches focusing on people at highest risk of anal cancer and integrated within existing care platforms, such as HIV care services, merit further exploration. More studies on anal cancer incidence rates by histological subtype and among different populations in sub-Saharan Africa are needed to inform the development of region-specific screening guidelines. In addition to targeted screening, universal gender-neutral HPV vaccination for individuals up to 26 years could also help reduce the incidence of anal cancer [18].

In conclusion, anal cancer rates in South African men and women are similar, but ∼4 times higher among PWH than those without HIV. Older individuals, people with a history of genital warts, and women with a prior diagnosis of cervical precancer are also at increased anal cancer risk. These individuals who are at increased anal cancer risk may benefit from prioritized access to screening for and treatment of precancerous anal lesions.

## Supplementary Material

ofaf537_Supplementary_Data

## Supplementary Data

Supplementary materials are available at *Open Forum Infectious Diseases* online. Consisting of data provided by the authors to benefit the reader, the posted materials are not copyedited and are the sole responsibility of the authors, so questions or comments should be addressed to the corresponding author.
