# Supplementary material for: Anal Cancer Incidence Rates Among Men and Women With and Without HIV in South Africa
Source: Open Forum Infect Dis. 2025 Sep 1;12(9):ofaf537. doi: 10.1093/ofid/ofaf537 (PMC12449734; doi:10.1093/ofid/ofaf537)
Supplement: ofaf537_Supplementary_Data [file ofaf537_supplementary_data.docx]

**Anal cancer incidence rates among men and women with and without HIV in South Africa**

**Supplementary Material**

**Tables**

- [**Supplementary Table 1.** Participant characteristics by sex 2](#_Toc187435450)
- [**Supplementary Table 2.** Characteristics of women with or without anal cancer. 3](#_Toc187435451)
- [**Supplementary Table 3.** Hazard ratios for the incidence of anal cancer, with time-at-risk starting one year earlier for HIV positive individuals. The adjusted models control for the risk factors listed in the table and for population group. 4](#_Toc187435452)
- [**Supplementary Table 4.** Hazard ratios for the incidence of anal cancer, with time-at-risk starting two years earlier for HIV positive individuals. The adjusted models control for the risk factors listed in the table and for population group. 5](#_Toc187435453)
- [**Supplementary Table 5.** Hazard ratios for the incidence of anal cancer**,** including individuals with only a single HIV marker in the HIV positive group. The adjusted models control for the risk factors listed in the table and for population group. 6](#_Toc187435454)
- [**Supplementary Table 6.** Hazard ratios for the incidence of anal cancer when including single-diagnosis cases. The adjusted models control for the risk factors listed in the table and for population group. 7](#_Toc187435455)

**Figures**

- [**Supplementary Figure 1.** Selection of study population. 8](#_Toc187435463)
- [**Supplementary Figure 2.** Hazard ratios over follow-up time, allowing for 1 degree of freedom in the interaction with follow-up-time, for all factors. A) Female vs. male, B) HIV positive vs. HIV negative, C) Age (reference=45-54), D) Calendar year (reference=2011-2013), E) History of genital warts vs. no history of genital warts, and F) History of other sexually transmitted infections (STIs) vs no history of other STIs. The models adjust for the risk factors displayed here and for population group. 9](#_Toc187435464)

**Supplementary Table 1.** Participant characteristics by sex.

|  | **Men**  **N = 511,198** | **Women**  **N = 557,717** | **Overall**  **N = 1,068,915** |
| --- | --- | --- | --- |
| HIV positive | 29,463 (6) | 40,522 (7) | 69,985 (7) |
| Median age at baseline^†^  [IQR] | 36.9 [26.8, 49.4] | 36.5 [26.5, 49.8] | 36.7 [26.7, 49.6] |
| Age group at baseline^†^ |  |  |  |
| 18-34 | 231,887 (45) | 259,987 (47) | 491,874 (46) |
| 35-44 | 109,425 (21) | 111,080 (20) | 220,505 (21) |
| 45-54 | 89,213 (18) | 90,839 (16) | 180,052 (17) |
| 55-64 | 51,938 (10) | 55,067 (10) | 107,005 (10) |
| 65+ | 28,735 (6) | 40,744 (7) | 69,479 (7) |
| Baseline year^†^ |  |  |  |
| 2011-2013 | 296,393 (58) | 311,781 (56) | 608,174 (57) |
| 2014-2016 | 83,885 (16) | 90,100 (16) | 173,985 (16) |
| 2017-2020 | 130,920 (26) | 155,836 (28) | 286,756 (27) |
| Genital warts^‡^ | 1,680 (<1) | 3,219 (1) | 4,899 (1) |
| Other STIs^‡^ | 16,491 (3) | 11,783 (2) | 28,274 (3) |

The results are reported as numbers and percentages if not otherwise stated.

^†^ Baseline refers to the start of time-at-risk.

^‡^ Diagnosis during or before time-at-risk.

IQR: interquartile range; STI: sexually transmitted infection.

**Supplementary Table 2.** Characteristics of women with or without anal cancer.

|  | **No anal cancer**  **N = 557,650** | **With anal cancer**  **N = 67** | **Overall**  **N = 557,717** |
| --- | --- | --- | --- |
| People with HIV | 40,511 (7) | 11 (16) | 40,522 (7) |
| Median age at baseline^†^  [IQR] | 36.5 [26.5, 49.8] | 57.2 [44.4, 67.5] | 36.5 [26.5, 49.8] |
| Age group at baseline^†^ |  |  |  |
| 18-34 | 259,980 (47) | 7 (10) | 259,987 (47) |
| 35-44 | 111,069 (20) | 11 (16) | 111,080 (20) |
| 45-54 | 90,824 (16) | 15 (22) | 90,839 (16) |
| 55-64 | 55,057 (10) | 10 (15) | 55,067 (10) |
| 65+ | 40,720 (7) | 24 (36) | 40,744 (7) |
| Baseline year^†^ |  |  |  |
| 2011-2013 | 311,732 (56) | 49 (73) | 311,781 (56) |
| 2014-2016 | 90,092 (16) | 8 (12) | 90,100 (16) |
| 2017-2020 | 155,826 (28) | 10 (15) | 155,836 (28) |
| Genital warts^‡^ | 3,217 (1) | 2 (3) | 3,219 (1) |
| Other STIs^‡^ | 11,782 (2) | 1 (2) | 11,783 (2) |
| Cervical pre-cancer^‡^ | 7,311 (1) | 4 (6) | 7,315 (1) |

The results are reported as numbers and percentages if not otherwise stated.

^†^ Baseline refers to the start of time-at-risk.

^‡^ Diagnosis during or before time-at-risk.

IQR: interquartile range; STI: sexually transmitted infection.

**Supplementary Table 3.** Hazard ratios with 95% confidence intervals for the incidence of anal cancer, with time-at-risk starting one year earlier for HIV positive individuals. The adjusted models control for the risk factors listed in the table and for population group.

|  | **Unadjusted** | **Adjusted** |
| --- | --- | --- |
| People with HIV vs. without HIV | 2.39 (1.50-3.82) | 4.69 (2.67-8.22) |
| Female vs. male | 1.06 (0.75-1.51) | 0.95 (0.67-1.36) |
| Age^†^ [years] |  |  |
| 18-34 | 0.14 (0.05-0.36) | 0.15 (0.06-0.41) |
| 35-44 | 0.60 (0.31-1.15) | 0.58 (0.30-1.11) |
| 45-54 | 1 | 1 |
| 55-64 | 1.58 (0.90-2.78) | 1.70 (0.96-2.99) |
| ≥65 | 4.65 (2.86-7.57) | 4.80 (2.86-8.06) |
| Calendar year^†^ |  |  |
| 2011-2013 | 1 | 1 |
| 2014-2016 | 1.50 (0.86-2.61) | 1.61 (0.91-2.85) |
| 2017-2020 | 2.18 (1.29-3.69) | 1.97 (1.13-3.41) |
| Genital warts, yes vs. no^†^ | 5.94 (1.88-18.75) | 7.15 (2.17-23.62) |
| Other sexually transmitted infections, yes vs. no^†^ | 1.00 (0.32-3.16) | 1.36 (0.41-4.45) |

^†^ Time-updated variables.

**Supplementary Table 4.** Hazard ratios with 95% confidence intervals for the incidence of anal cancer, with time-at-risk starting two years earlier for individuals with HIV. The adjusted models control for the risk factors listed in the table and for population group.

|  | **Unadjusted** | **Adjusted** |
| --- | --- | --- |
| People with HIV vs. without HIV | 2.33 (1.47-3.68) | 4.70 (2.71-8.16) |
| Female vs. male | 1.08 (0.76-1.53) | 0.97 (0.68-1.38) |
| Age^†^ [years] |  |  |
| 18-34 | 0.14 (0.05-0.36) | 0.15 (0.06-0.41) |
| 35-44 | 0.64 (0.34-1.21) | 0.61 (0.32-1.16) |
| 45-54 | 1 | 1 |
| 55-64 | 1.59 (0.90-2.78) | 1.71 (0.97-3.01) |
| ≥65 | 4.67 (2.87-7.60) | 4.85 (2.89-8.15) |
| Calendar year^†^ |  |  |
| 2011-2013 | 1 | 1 |
| 2014-2016 | 1.49 (0.85-2.61) | 1.63 (0.92-2.89) |
| 2017-2020 | 2.21 (1.31-3.75) | 2.04 (1.18-3.54) |
| Genital warts, yes vs. no^†^ | 5.81 (1.84-18.35) | 7.00 (2.12-23.07) |
| Other sexually transmitted infections, yes vs. no^†^ | 0.98 (0.31-3.09) | 1.33 (0.41-4.36) |

^†^ Time-updated variables.

**Supplementary Table 5.** Hazard ratios with 95% confidence intervals for the incidence of anal cancer**,** including individuals with only a single HIV marker as people with HIV. The adjusted models control for the risk factors listed in the table and for population group.

|  | **Unadjusted** | **Adjusted** |
| --- | --- | --- |
| People with HIV vs. without HIV | 2.07 (1.25-3.42) | 3.88 (2.15-7.01) |
| Female vs. male | 1.09 (0.76-1.55) | 0.97 (0.68-1.39) |
| Age^†^ [years] |  |  |
| 18-34 | 0.15 (0.06-0.40) | 0.17 (0.06-0.46) |
| 35-44 | 0.62 (0.31-1.21) | 0.60 (0.31-1.19) |
| 45-54 | 1 | 1 |
| 55-64 | 1.72 (0.97-3.06) | 1.79 (1.00-3.20) |
| ≥65 | 5.06 (3.06-8.36) | 4.91 (2.89-8.34) |
| Calendar year^†^ |  |  |
| 2011-2013 | 1 | 1 |
| 2014-2016 | 1.34 (0.78-2.32) | 1.44 (0.81-2.56) |
| 2017-2020 | 1.69 (1.01-2.85) | 1.70 (0.98-2.97) |
| Genital warts, yes vs. no^†^ | 5.96 (1.88-18.94) | 7.93 (2.40-26.25) |
| Other sexually transmitted infections, yes vs. no^†^ | 1.00 (0.32-3.16) | 1.45 (0.44-4.77) |

^†^ Time-updated variables.

**Supplementary Table 6.** Hazard ratios for the incidence of anal cancer when including single-diagnosis cases. The adjusted models control for the risk factors listed in the table and for population group.

|  | **Unadjusted** | **Adjusted** |
| --- | --- | --- |
| People with HIV vs. without HIV | 1.66 (1.10-2.52) | 2.91 (1.82-4.66) |
| Female vs. male | 0.82 (0.63-1.07) | 0.74 (0.57-0.96) |
| Age^†^ [years] |  |  |
| 18-34 | 0.16 (0.08-0.33) | 0.18 (0.09-0.37) |
| 35-44 | 0.69 (0.43-1.11) | 0.70 (0.43-1.13) |
| 45-54 |  |  |
| 55-64 | 2.10 (1.40-3.15) | 2.10 (1.39-3.15) |
| ≥65 | 4.35 (2.98-6.34) | 3.95 (2.66-5.85) |
| Calendar year^†^ |  |  |
| 2011-2013 |  |  |
| 2014-2016 | 1.07 (0.71-1.60) | 1.12 (0.74-1.69) |
| 2017-2020 | 1.60 (1.09-2.33) | 1.41 (0.95-2.10) |
| Genital warts, yes vs. no^†^ | 4.34 (1.61-11.68) | 6.42 (2.31-17.84) |
| Other sexually transmitted infections, yes vs. no^†^ | 0.73 (0.27-1.96) | 1.11 (0.40-3.06) |

^†^ Time-updated variables.


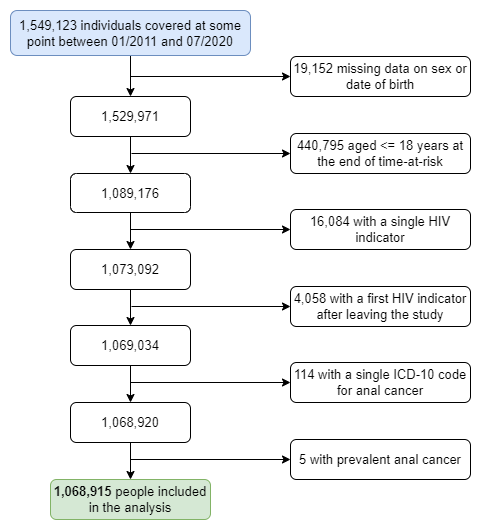


**Supplementary Figure 1.** Selection of study population.

**
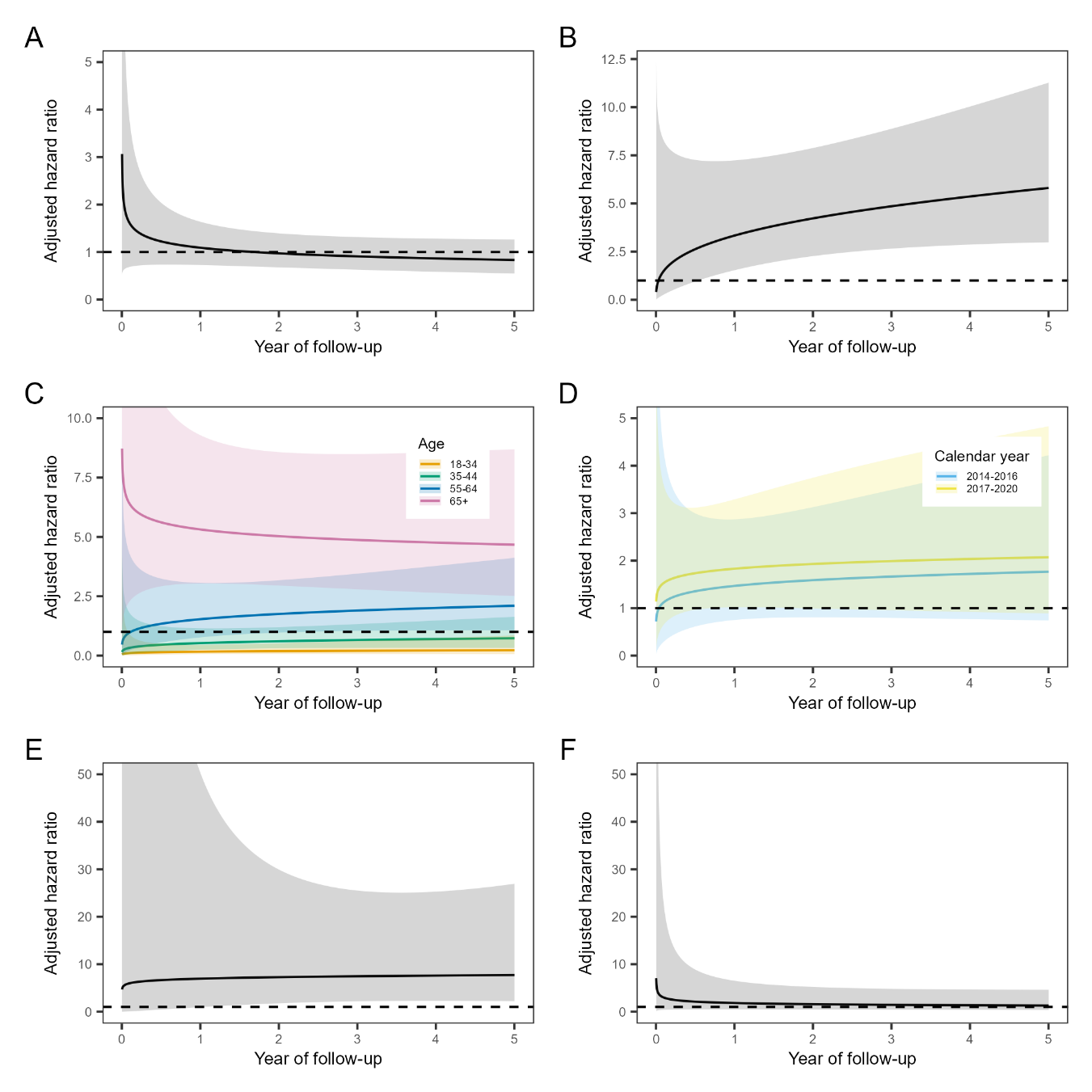
**

**Supplementary Figure 2.** Hazard ratios over follow-up time, allowing for 1 degree of freedom in the interaction with follow-up-time, for all factors. A) Female vs. male, B) HIV positive vs. HIV negative, C) Age (reference=45-54), D) Calendar year (reference=2011-2013), E) History of genital warts vs. no history of genital warts, and F) History of other sexually transmitted infections (STIs) vs no history of other STIs. The models adjust for the risk factors displayed here and for population group.
